# Cockayne syndrome B protein regulates recruitment of the Elongin A ubiquitin ligase to sites of DNA damage

**DOI:** 10.1074/jbc.C117.777946

**Published:** 2017-03-14

**Authors:** Juston C. Weems, Brian D. Slaughter, Jay R. Unruh, Stefan Boeing, Shawn M. Hall, Merry B. McLaird, Takashi Yasukawa, Teijiro Aso, Jesper Q. Svejstrup, Joan W. Conaway, Ronald C. Conaway

**Affiliations:** From the ‡ Stowers Institute for Medical Research, Kansas City, Missouri 64110,; the § Mechanisms of Transcription Laboratory, The Francis Crick Institute, Clare Hall Laboratories, South Mimms EN6 3LD, United Kingdom,; the ‖ Department of Biochemistry and Molecular Biology, University of Kansas Medical Center, Kansas City, Kansas 66160, and; the ¶ Department of Functional Genomics, Kochi Medical School, Kohasu, Oko-cho, Nankoku, Kochi 783-8505, Japan

**Keywords:** DNA repair, E3 ubiquitin ligase, nucleotide excision repair, RNA polymerase II, transcription

## Abstract

Elongin A performs dual functions as the transcriptionally active subunit of RNA polymerase II (Pol II) elongation factor Elongin and as the substrate recognition subunit of a Cullin-RING E3 ubiquitin ligase that ubiquitylates Pol II in response to DNA damage. Assembly of the Elongin A ubiquitin ligase and its recruitment to sites of DNA damage is a tightly regulated process induced by DNA-damaging agents and α-amanitin, a drug that induces Pol II stalling. In this study, we demonstrate (i) that Elongin A and the ubiquitin ligase subunit CUL5 associate in cells with the Cockayne syndrome B (CSB) protein and (ii) that this interaction is also induced by DNA-damaging agents and α-amanitin. In addition, we present evidence that the CSB protein promotes stable recruitment of the Elongin A ubiquitin ligase to sites of DNA damage. Our findings are consistent with the model that the Elongin A ubiquitin ligase and the CSB protein function together in a common pathway in response to Pol II stalling and DNA damage.

## Introduction

Elongin A was originally discovered as the transcriptionally active subunit of RNA polymerase II (Pol II)^3^ transcription factor Elongin (SIII), which stimulates the overall rate of Pol II elongation through a direct interaction with the enzyme (1, 2). Elongin is composed of Elongin A and a heterodimeric submodule composed of the Elongin B and C proteins, which bind to a short Elongin A sequence motif referred to as the BC-box and potently activate Elongin A transcriptional activity (3–6).

The Elongin heterotrimer was subsequently found to assemble with Cullin family member CUL5 and RING finger protein RBX (7, 8) to form an E3 ubiquitin ligase that targets the RPB1 subunit of Pol II for ubiquitylation and degradation by the proteasome in cells subjected to UV or oxidative DNA damage (9–12). Based on the observations (i) that the Elongin A ubiquitin ligase targets RPB1 for ubiquitylation *in vitro* (11, 12) and (ii) that mutations in subunits of the Elongin A ubiquitin ligase result in reduced or delayed ubiquitylation and degradation of RPB1 after DNA damage (9–11, 13), the ligase has been proposed to play a critical role in the removal of Pol II stalled at sites of DNA damage. Notably, the Elongin A ubiquitin ligase was among the founding members of the large BC-box (SOCS-box) family of Cullin-RING E3 ubiquitin ligases (reviewed in Ref. 14). Members of this family include a BC-box-containing substrate recognition subunit that binds to the Elongin BC heterodimer, which in turn acts as an adaptor to link the BC-box protein to a submodule composed of a Cullin protein (CUL2 or CUL5) and one of two RBX paralogs (RBX1 or RBX2) (7, 8, 15).

We wish to understand how the assembly and activity of Elongin A ubiquitin ligase are regulated. In a previous study, we obtained evidence consistent with the model (i) that assembly of the ligase is a tightly regulated process that can be rapidly induced by treatment of cells with a large variety of DNA-damaging agents or with drugs that induce Pol II stalling, and (ii) that once assembled, the ligase is rapidly recruited to sites of DNA damage (16). In a recent proteomic screen, Boeing *et al.* (17) identified Elongin A as one of a number of proteins that exhibit increased interaction with the Cockayne syndrome B (CSB) protein after induction of DNA damage by UV irradiation. Cockayne syndrome is a neurodevelopmental disorder in which affected individuals exhibit neural and growth abnormalities, progeroid features, and sun sensitivity (18). It is caused by mutations in either the *ERCC6* or *ERCC8* genes, encoding CSB or the Cockayne Syndrome A (CSA) protein, respectively.

The CSB protein is a SNF2 family ATPase. Mutation or deletion of the gene encoding CSB alters the expression of a large number of genes, many of which have roles in neuronal differentiation or function (19–22). In addition, the CSB protein regulates transcription-coupled nucleotide excision repair, base excision repair, and repair of double-strand breaks (23–27). Although the precise mechanisms by which CSB functions in these processes have not been elucidated, it has been proposed to function as a sensor for DNA damage and/or Pol II stalled at DNA damage sites (28–31).

In this study, we describe experiments exploring the potential connection between the Elongin A ubiquitin ligase and the CSB protein. Evidence from these experiments has revealed (i) that association of the Elongin A ubiquitin ligase with the CSB protein is a tightly regulated process that is rapidly induced by treatment of cells with DNA-damaging agents or inhibitors of Pol II elongation and (ii) that the CSB protein is required for stable recruitment of the Elongin A ubiquitin ligase to DNA damage sites.

## Results and discussion

**Figure 1.**
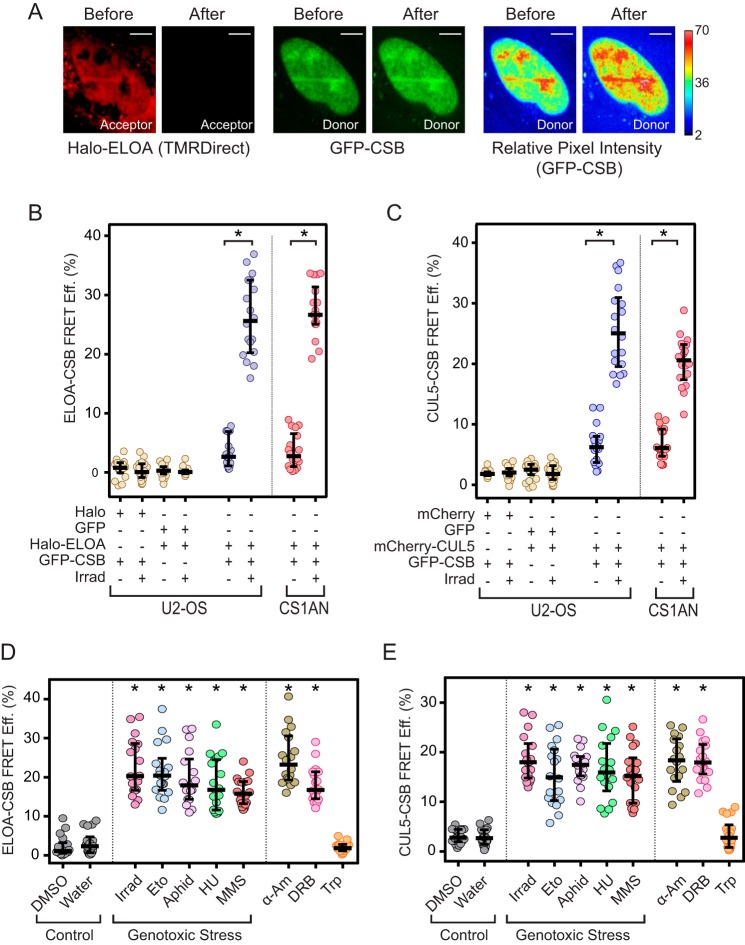
**Regulated interaction of CSB with Elongin A and CUL5.** *A*, representative images of acceptor and donor fluorescence in a CS1ANsv cell transiently expressing Halo-Elongin A labeled with TMRDirect^TM^ (*left*) and GFP-CSB (*middle*), before and after acceptor photobleaching. On the *right* are heat maps showing relative donor pixel intensity before and after acceptor photobleaching. *Scale bars*, 8 μm. *B* and *C*, AP-FRET efficiency (*FRET Eff.*) in Hoechst-sensitized U2-OS or CS1ANsv cells transiently expressing the indicated proteins. Cells were subjected or not to laser microirradiation (*Irrad*). *, *p* < 10^−4^ (unpaired *t* test). *Halo-ELOA*, Halo-tagged Elongin A. *D* and *E*, AP-FRET efficiency in Hoechst-sensitized CS1ANsv-GFP-CSB cells transiently expressing Halo-Elongin A or mCherry-CUL5 and subjected to treatment with the indicated compound for 1 h or to laser microirradiation. *Eto*, etoposide; *aphid*, aphidicolin; *HU*, hydroxyurea; *MMS*, methyl methanesulfonate; α*-Am*, α-amanitin; *Trp*, triptolide. Graphs show values from individual cells with median and interquartile ranges. *n* = 18 cells (6 cells from each of 3 independent experiments). *, *p* < 10^−4^ as compared with water control (Dunnett's multiple-comparison test after analysis of variance).

To explore the connection between the Elongin A ubiquitin ligase and the CSB protein in living cells, we exploited acceptor photobleaching fluorescence resonance energy transfer (AP-FRET). During our FRET assays, energy is transferred from a green donor fluorophore to a red acceptor fluorophore. As a result of the energy transfer, the emission of the donor is quenched and that of the acceptor is enhanced in a strongly distance-dependent fashion; for typical fluorescent proteins, FRET occurs only at distances less than 100 Å (32, 33). In AP-FRET, FRET efficiency is determined by comparing the donor fluorescence emission before acceptor photobleaching (when energy can be transferred to acceptor molecules) with donor emission after acceptor photobleaching (when the acceptor can no longer absorb energy emitted from the donor) (Fig. 1*A*). We previously employed AP-FRET successfully to show that interaction of Elongin A with CUL5 to form the Elongin A ubiquitin ligase is tightly regulated in living cells (16).

Interactions between CSB and subunits of the Elongin A ubiquitin ligase were monitored in human bone osteosarcoma U2-OS cells or in CS1ANsv cells. CS1ANsv cells are SV40 immortalized human fibroblasts derived from a Cockayne syndrome patient lacking functional CSB; they express a 336-amino acid, N-terminal fragment of CSB truncated upstream of the SNF2 ATPase domain (23, 34). CS1ANsv cells exhibit transcription and DNA repair defects that can be rescued by exogenous expression of wild-type CSB (for example, see Refs. 20 and 23). In initial experiments, Hoechst-sensitized cells transiently expressing GFP-tagged CSB (GFP-CSB) and Halo-tagged Elongin A (Halo-EloA) labeled with a cell-permeable rhodamine 110 derivative were subjected or not to UV laser microirradiation. UV laser microirradiation of Hoechst-sensitized cells gives rise to DNA lesions, including cyclobutane pyrimidine dimers and pyrimidine-pyrimidone (6-4) photoproducts, oxidative lesions, and single- and double-stranded breaks (35, 36). In control assays, we detected little or no FRET between Halo-Elongin A and free GFP or between GFP-CSB and free Halo tag in cells that had or had not been subjected to laser microirradiation (Fig. 1*B*). In contrast, microirradiation induced strong AP-FRET signals between Halo-Elongin A and GFP-CSB in both U2-OS and CS1ANsv cells. In parallel experiments, substantial AP-FRET signals were also detected following laser microirradiation of Hoechst-sensitized cells expressing mCherry-CUL5 and GFP-CSB, but not between mCherry-CUL5 and free GFP or GFP-CSB and free mCherry (Fig. 1*C*). Similar increases in AP-FRET signals between CSB and Elongin A or CUL5 were observed when experiments were performed using CS1ANsv cells stably expressing GFP-CSB (CS1ANsv-GFP-CSB cells) and either transiently expressed Halo-Elongin A (Fig. 1*B*) or transiently expressed mCherry-CUL5 (Fig. 1*C*).

Binding of Elongin A to CUL5 to form the Elongin A ubiquitin ligase can be provoked not only by UV irradiation of Hoechst-sensitized cells, but also by treatment of cells with a collection of DNA-damaging agents that give rise to single- and double-strand DNA breaks. In addition, treatment of cells with α-amanitin or 5,6-dichloro-1-β-d-ribofuranosylbenzimidazole (DRB), which both induce Pol II stalling without DNA damage, can drive assembly of the Elongin A ubiquitin ligase (16). To determine whether these same treatments induce binding of CSB to the Elongin A ubiquitin ligase, we measured AP-FRET between GFP-CSB and Halo-Elongin A or mCherry CUL5 in CS1ANsv-GFP-CSB (Fig. 1, *D* and *E*). Treatment of cells with the topoisomerase 1 inhibitor camptothecin, the topoisomerase 2 inhibitor etoposide, the DNA-alkylating agent methyl methanesulfonate, and the DNA replication inhibitors aphidicolin and hydroxyurea all induced AP-FRET between CSB and both Elongin A and CUL5. In addition, the Pol II elongation inhibitors α-amanitin and DRB induced AP-FRET between CSB and Elongin A or CUL5, whereas triptolide, which blocks Pol II initiation (37, 38), did not. Taken together, these findings argue that CSB interacts with both Elongin A and the ubiquitin ligase subunit CUL5 in response to treatments that cause DNA damage or Pol II stalling.

**Figure 2.**
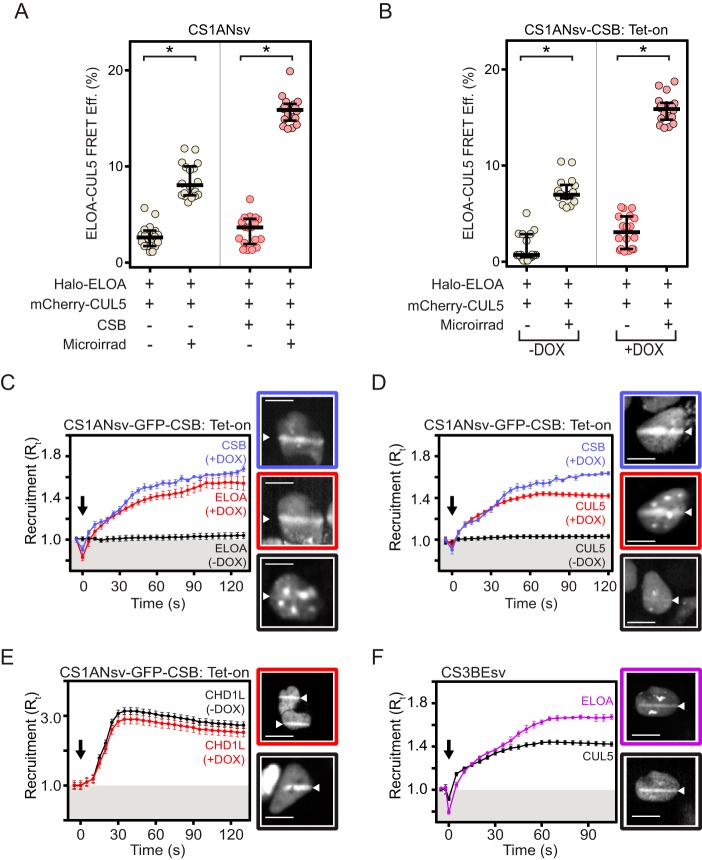
**CSB-dependent recruitment of Elongin A and CUL5 to localized DNA damage.** *A* and *B*, AP-FRET efficiency (*FRET Eff.*) in CS1ANsv cells (*A*) or CS1ANsv-CSB Tet-on cells (*B*) transiently expressing the indicated proteins and subjected or not to laser microirradiation (*Microirrad*) as indicated. Graphs show AP-FRET values from individual cells with median and interquartile ranges; *n* = 18 cells (6 cells from each of 3 independent experiments). *, *p* < 10^−4^. *Halo-ELOA*, Halo-tagged Elongin A. *C* and *D*, recruitment of GFP-CSB and either Halo-Elongin A (*C*) or mCherry-CUL5 (*D*) in CS1ANsv-GFP-CSB TET-on cells. Induction of CSB expression results in a significant increase in Elongin A and CUL5 recruitment (*p* < 10^−4^, based on comparisons of last 10 time points in unpaired *t* tests). *E*, recruitment of Halo-CHD1L in CS1ANsv-GFP-CSB Tet-on cells. *F*, recruitment of Halo-Elongin A and mCherry-CUL5 in CS3BEsv cells. Cells were imaged every second, and intensity values were binned over 5-s intervals. Microirradiation was initiated at time *t* = 0 (*black arrow*). Graphs show mean ± S.E., *n* = 18 cells (6 cells from each of 3 independent experiments). Where indicated, cells were treated with doxycycline (*DOX*) to induce CSB expression. Included in *panels C*, *D*, *E*, and *F* are representative images of cells subjected to microirradiation; *white triangles* indicate regions of microirradiation. *Scale bars*, 8 μm. For *panels C* and *D*, images boxed in *blue* show GFP-CSB fluorescence in doxycycline-treated cells, and images boxed in *red* show Halo-Elongin A or mCherry-CUL5 fluorescence, respectively, in the same cells; images boxed in *black* show either Halo-Elongin A or mCherry-CUL5 fluorescence in cells grown without doxycycline. For *panel E*, images boxed in *red* or *black* show Halo-CHD1L fluorescence in cells grown in the presence or absence of doxycycline, respectively. For *panel F*, images boxed in *purple* and *black* show Halo-Elongin A and mCherry CUL5 fluorescence, respectively, in the same CS3BEsv cell.

We next asked whether the CSB protein might play a role in assembly of the Elongin A ubiquitin ligase, its recruitment to sites of DNA damage, or both processes. To determine whether CSB contributes to assembly of the Elongin A ubiquitin ligase, we measured AP-FRET between Halo-Elongin A and mCherry-CUL5 in CSB-deficient CS1ANsv cells transiently transfected with a plasmid encoding CSB or with empty vector (Fig. 2*A*) or in a CS1ANsv cell line (CS1AN-CSB Tet-on) stably expressing untagged CSB under control of a doxycycline-inducible promoter (Fig. 2*B*). The Elongin A-CUL5 AP-FRET signal was increased following UV-microirradiation of cells lacking wild-type CSB but was further enhanced upon expression of CSB. Thus, wild-type CSB can contribute to, but is not essential for, DNA damage-induced assembly of the Elongin A ubiquitin ligase.

In contrast, CSB plays a critical role in stable recruitment of both Elongin A and CUL5 to sites of localized DNA damage. We previously observed that both Elongin A and CUL5 are rapidly recruited to regions of localized DNA damage induced by UV laser microirradiation of the nuclei of Hoechst-sensitized cells (16). To test the contribution of CSB to this recruitment, CS1AN cells carrying doxycycline-inducible GFP-CSB were grown in the presence or absence of doxycycline, transfected with plasmids encoding Halo-Elongin A or mCherry-CUL5, and subjected to laser microirradiation to create a stripe of localized DNA damage in cell nuclei. Included in Fig. 2 are representative images of cells taken 1 min after laser microirradiation; in these images, microirradiated regions are indicated by *white arrowheads*. The kinetics of accumulation of fluorescently labeled proteins at these sites was determined by imaging cells over a period of 2 min after laser microirradiation. The recruitment ratio (*R_t_*) is a measure of fluorescence intensity at the microirradiated region relative to total nuclear fluorescence at each time point; an *R_t_* greater than one indicates an accumulation of fluorescently labeled protein at the microirradiated region.

Consistent with previous results (29, 39, 40), in doxycycline-treated cells, GFP-CSB was rapidly recruited to regions of DNA damage (Fig. 2, *C* and *D*, images outlined in *blue*). Under these conditions, Elongin A and CUL5 also accumulated at regions of laser-induced DNA damage (images outlined in *red*). Notably, CSB, Elongin A, and CUL5 all accumulated at these regions with very similar kinetics. In cells that were grown in the absence of doxycycline and lacked functional CSB, we observed a very slight enrichment of Elongin A and CUL5 at sites of microirradiation (Fig. 2, *C* and *D*, see images outlined in *black*); however, there was much less accumulation of these proteins than in cells expressing wild-type CSB.

The very small but detectable level of Elongin A or CUL5 recruitment observed in cells grown in the absence of doxycycline cannot be explained by leaky expression of CSB, because similar results were obtained in experiments measuring recruitment to regions of laser-induced DNA damage in CS1ANsv cells transiently transfected or not with GFP-CSB (supplemental Fig. 1). Nevertheless, we cannot exclude the possibility that the residual Elongin A and CUL5 recruitment seen in the absence of exogenous wild-type CSB depends on the N-terminal CSB fragment expressed by CS1ANsv cells.

Our observation that Elongin A and CUL5 recruitment is defective in the absence of wild-type CSB does not reflect a general failure to recruit DNA repair proteins to regions of localized DNA damage in these cells, because we observed that recruitment of the poly(ADP-ribose) polymerase (PARP)-activated chromatin-remodeling enzyme CHD1L (41, 42) was independent of CSB expression (Fig. 2*E*). Finally, we note that Elongin A (*purple*) and CUL5 (*black*) recruitment to sites of laser-induced DNA damage was not impaired in CS3BEsv cells (Fig. 2*F*), which are SV40 immortalized human fibroblasts derived from a Cockayne syndrome patient lacking functional CSA but expressing wild-type CSB.

**Figure 3.**
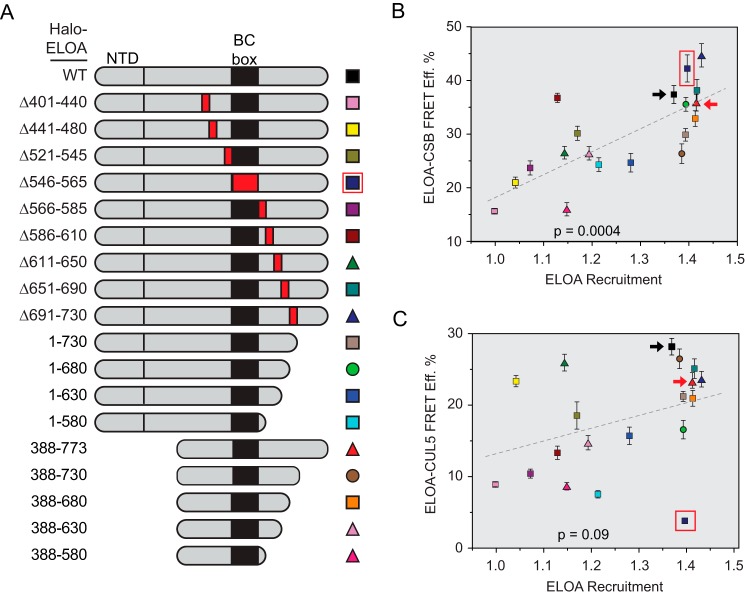
**Effects of Elongin A mutations on CSB or CUL5 binding and recruitment to DNA damage.** *A*, schematic representation of wild-type and mutant Elongin As. *Red boxes* indicate positions of internal deletions. *Halo-ELOA*, Halo-tagged Elongin A; *NTD*, N-terminal domain. *B* and *C*, Elongin A-CSB or Elongin A-CUL5 AP-FRET efficiency (*FRET Eff.*) *versus* Elongin A recruitment. AP-FRET efficiency is expressed as mean ± S.E.; Elongin A recruitment is the *R_t_* value measured at 30 s after microirradiation. *p* values are for comparison of the slopes of the linear regressions to a zero slope. *Black arrows* highlight wild-type Elongin A, *red arrows* highlight the Elongin A(388–773) mutant, which lacks the TFIIS- and MED26-like N-terminal domain, and a *red box* highlights Elongin A(Δ546–565), which lacks the BC-box.

The data presented thus far demonstrate (i) that interaction of CSB with the Elongin A ubiquitin ligase can be provoked by laser microirradiation and other DNA-damaging agents, (ii) that CSB and the Elongin A ubiquitin ligase are recruited to sites of laser-induced DNA damage with similar kinetics, and (iii) that CSB is needed for stable recruitment of the Elongin A ubiquitin ligase to DNA damage sites. It does not, however, provide insight into whether the Elongin A-CSB interaction is needed for CSB-dependent recruitment. To explore the relationship between CSB-dependent recruitment of Elongin A to regions of DNA damage and DNA damage-induced CSB-Elongin A interaction, we assayed an extensive series of Halo-Elongin A N-terminal, C-terminal, and internal deletion mutants (Fig. 3*A*) for their abilities to interact with CSB in AP-FRET experiments (supplemental Fig. 2*A*) and to accumulate at sites of laser microirradiation (supplemental Fig. 2*B*). As shown in Fig. 3*B*, we observed a significant correlation (*p* = 0.0004) between the abilities of the Elongin A mutants to interact with CSB and to be recruited to DNA damage regions, consistent with the model that the interaction between Elongin A and CSB contributes to CSB-dependent Elongin A recruitment. In contrast, and consistent with previous results obtained with a more limited series of Elongin A mutants (16), the correlation between their abilities to assemble with CUL5 to form the Elongin A ubiquitin ligase and to be recruited to damage regions was less significant (Fig. 3*C* and supplemental Fig. 2*C*). Of note, deletion of the Elongin A BC-box (outlined in *red* in Fig. 3, *B* and *C*) abrogates the Elongin A-CUL5 interaction without affecting either the Elongin A-CSB interaction or Elongin A recruitment to DNA damage, indicating that the CSB-Elongin A interaction and CSB-dependent recruitment of Elongin A to DNA damage can occur without prior assembly of the Elongin A ubiquitin ligase. In addition, Elongin A's conserved N-terminal domain, which resembles the N-terminal domains of the transcription elongation factor TFIIS and Mediator subunit MED26, is dispensable for its interactions with CSB and CUL5 and its recruitment to regions of DNA damage (Fig. 3, *B* and *C*, compare wild-type Elongin A (*black arrows*) with Elongin A(388–773) (*red arrows*)).

In summary, here we have shown that the CSB protein (i) interacts with Elongin A and the Elongin A ubiquitin ligase in response to DNA damage and Pol II stalling, (ii) enhances, but is not essential for, assembly of the Elongin A ubiquitin ligase provoked by DNA damage and Pol II stalling, and (iii) promotes stable recruitment of Elongin A and the Elongin A ubiquitin ligase to regions of localized DNA damage. Although the precise mechanism of action of the CSB protein in repair of DNA damage remains mysterious, our results argue that at least one function of CSB is to expedite recruitment of the Elongin A ubiquitin ligase to sites of DNA damage for ubiquitylation and proteasomal removal of stalled Pol II. Our findings are consistent with previous proposals that CSB functions as a sensor of stalled Pol II to initiate the DNA repair process (28–31).

## Experimental procedures

### Materials

HaloTag® R110Direct^TM^ Ligand G3221 and HaloTag® TMRDirect^TM^ Ligand G2991 were purchased from Promega. Hoechst solution (33258; used at 1:1000 dilution), aphidicolin (A-4487; used at 4 μm), hydroxyurea (H-8627; used at 200 μm), methyl methanesulfonate (129925; used at 0.1 μm), hydrogen peroxide (16911; used at 300 μm), DRB (D1916, used at 25 μm), and triptolide (T-3652, used at 10 μm) were all from Sigma. α-Amanitin (used at 10 μm) was purchased from Sigma (A2263) or EMD/Millipore (129741). Etoposide (341205; used at 100 μm) was purchased from Millipore, Calbiochem. FuGENE HD transfection reagent was obtained from Promega.

### Cell culture and stable cell lines

CS1ANsv cells (43), CS1ANsv cells constitutively expressing CSB (CS1ANsv-CSB) or GFP-CSB (CS1ANsv-GFP-CSB) at near physiological levels (29), and CS3BEsv cells (43) were cultured in phenol red-free DMEM. U2-OS cells (ATCC HTB-96) were cultured in phenol red-free McCoy's medium (Gibco, Invitrogen), at 37 °C in 5% CO_2_. DMEM and McCoy's media were supplemented with 5% GlutaMAX, 10% charcoal-stripped One Shot^TM^ fetal bovine serum (Gibco, Life Technologies), 100 units/ml penicillin, and 100 μg/ml streptomycin (Gibco, Life Technologies). CS1AN-CSB Tet-on cells (20) were cultured in phenol red-free DMEM containing 10% (v/v) Tet system-approved fetal bovine serum (Clontech) at 37 °C with 5% CO_2_ without antibiotics. Where indicated, cells were treated with 0.5 μg/ml doxycycline to induce CSB expression 24 h prior to use for transfection.

### Plasmids

Plasmids encoding Halo-tagged versions of wild-type and mutant rat Elongin A (GenBank^TM^ accession number AAA82095), mCherry-tagged CUL5 (mCherry-CUL5), human Elongins B and C, and FLAG-Halo-tagged CHD1L were generated as described (16). Plasmids encoding wild-type and GFP-tagged CSB were generated as described (44).

### Microirradiation, live imaging, AP-FRET, and image analysis

Time-lapse movies, UV micro-irradiation, and AP-FRET measurements were performed as described previously (16). Prior to microirradiation and/or AP-FRET, CS1AN or U2-OS cells were plated at 50–60% confluence in MatTek glass bottom dishes (35 mm, No. 2 14-mm diameter glass) and transfected using FuGENE HD and plasmids encoding Halo-Elongin A (100 ng as donor, 400 ng as acceptor), mCherry-CUL5 (100 ng or 400 ng), and GFP-CSB (100 ng) as indicated, together with plasmids encoding Elongin B (100 ng) and Elongin C (100 ng). To label Halo-tagged proteins with rhodamine 110 or TMRDirect^TM^ in living cells, medium was changed after 24 h, HaloTag® R110Direct^TM^ (when Elongin A was the FRET donor) or TMRDirect^TM^ (when Elongin A was the FRET acceptor) ligand was added to a final concentration of 100 nm, and cells were allowed to incubate overnight without washing as directed in the manufacturer's protocol. Cells were stained for 30 min with Hoechst dye to mark nuclei and/or sensitize cells to UV irradiation 24 h after transfection, and then allowed to recover for 5 min before AP-FRET measurements.

Values for normalized recruitment after microirradiation (*R_t_*) were calculated using the equation *R_t_* = (*I_t_/T_t_*)*/*(*I*_0_*/T*_0_). *I*_0_ and *T*_0_ are the average fluorescence intensities of the microirradiated and total nuclear region, respectively, averaged over the pre-irradiation time period. *I_t_* is the fluorescence intensity of the microirradiated stripe as a function of time and was measured as the average intensity of a manually selected region corresponding to the visible bleached region immediately after microirradiation. *T_t_* (total nuclear fluorescence intensity) was measured in the same way selecting the nuclear boundary. For measurements of AP-FRET, a sequence of at least three images of each region of interest was collected before and after photobleaching of the mCherry or TMRDirect^TM^ photoacceptor with 15 iterations of 100% 561-nm laser power. FRET efficiencies (*E*) were calculated using the equation *E* = 1 − 〈*I*_before_〉/〈*I*_after_〉, where the brackets represent a temporal average, and *I*_before_ and *I*_after_ refer to the donor fluorescence intensity before and after acceptor photobleaching. Statistical analyses were performed in GraphPad Prism 6.

### Original data

Original data underlying this paper can be accessed from the Stowers Original Data Repository at http://www.stowers.org/research/publications/LIBPB-1068. Please note that the JBC is not responsible for the long-term archiving and maintenance of this site or any other third-party-hosted site.

## Author contributions

J. C. W., J. W. C., and R. C. C. conceived and designed the study, analyzed data, and wrote the manuscript. J. C. W. performed most of the experiments. B. R. S. and J. R. U. assisted in assay design and interpretation. M. B. M and S. M. H. constructed vectors used for expression of mutant proteins. T. Y. and T. A. provided key reagents. S. B. and J. Q. S. contributed to experimental conception and design and provided key reagents. All authors approved the manuscript.

## Supplementary Material

Supplemental Data

## Footnotes

3 The abbreviations used are:

Pol II: RNA polymerase II
CSA: Cockayne syndrome A
CSB: Cockayne syndrome B
AP-FRET: acceptor photobleaching fluorescence resonance energy transfer
DRB: 5,6-dichloro-1-β-d-ribofuranosylbenzimidazole
Tet: Tetracycline.

## References

[1] Bradsher J. N., Jackson K. W., Conaway R. C., and Conaway J. W. (1993) RNA polymerase II transcription factor SIII: I. Identification, purification, and properties. J. Biol. Chem. 268, 25587–25593 8244996

[2] Bradsher J. N., Tan S., McLaury H.-J., Conaway J. W., and Conaway R. C. (1993) RNA polymerase II transcription factor SIII: II. Functional properties and role in RNA chain elongation. J. Biol. Chem. 268, 25594–25603 7503981

[3] Aso T., Lane W. S., Conaway J. W., and Conaway R. C. (1995) Elongin (SIII): a multisubunit regulator of elongation by RNA polymerase II. Science 269, 1439–1443 7660129 10.1126/science.7660129

[4] Garrett K. P., Aso T., Bradsher J. N., Foundling S. I., Lane W. S., Conaway R. C., and Conaway J. W. (1995) Positive regulation of general transcription SIII by a tailed ubiquitin homolog. Proc. Natl. Acad. Sci. U.S.A. 92, 7172–7176 7638163 10.1073/pnas.92.16.7172 PMC41301

[5] Aso T., Haque D., Barstead R. J., Conaway R. C., and Conaway J. W. (1996) The inducible Elongin A elongation activation domain: structure, function, and interaction with the Elongin BC complex. EMBO J. 15, 5557–5566 8896449 PMC452300

[6] Garrett K. P., Tan S., Bradsher J. N., Lane W. S., Conaway J. W., and Conaway R. C. (1994) Molecular cloning of an essential subunit of RNA polymerase II elongation factor SIII. Proc. Natl. Acad. Sci. U.S.A. 91, 5237–5241 8202474 10.1073/pnas.91.12.5237 PMC43969

[7] Kamura T., Burian D., Yan Q., Schmidt S. L., Lane W. S., Querido E., Branton P. E., Shilatifard A., Conaway R. C., and Conaway J. W. (2001) MUF1, A novel Elongin BC-interacting leucine-rich repeat protein that can assemble with Cul5 and Rbx1 to reconstitute a ubiquitin ligase. J. Biol. Chem. 276, 29748–29753 11384984 10.1074/jbc.M103093200

[8] Kamura T., Maenaka K., Kotoshiba S., Matsumoto M., Kohda D., Conaway R. C., Conaway J. W., and Nakayama K. I. (2004) VHL-box and SOCS-box domains determine binding specificity for Cul2-Rbx1 and Cul5-Rbx2 modules of ubiquitin ligases. Genes Dev. 18, 3055–3065 15601820 10.1101/gad.1252404 PMC535916

[9] Ribar B., Prakash L., and Prakash S. (2006) Requirement of *ELC1* for RNA polymerase II polyubiquitylation and degradation in response to DNA damage in *Saccharomyces cerevisiae*. Mol. Cell. Biol. 26, 3999–4005 16705154 10.1128/MCB.00293-06 PMC1489084

[10] Ribar B., Prakash L., and Prakash S. (2007) *ELA1* and *CUL3* are required along with *ELC1* for RNA polymerase II polyubiquitylation and degradation in DNA-damaged yeast cells. Mol. Cell Biol. 27, 3211–3216 17296727 10.1128/MCB.00091-07 PMC1899920

[11] Yasukawa T., Kamura T., Kitajima S., Conaway R. C., Conaway J. W., and Aso T. (2008) Mammalian Elongin A complex mediates DNA-damage-induced ubiquitylation and degradation of Rpb1. EMBO J. 27, 3256–3266 19037258 10.1038/emboj.2008.249 PMC2609743

[12] Harreman M., Taschner M., Sigurdsson S., Anindya R., Reid J., Somesh B., Kong S. E., Banks C. A., Conaway R. C., Conaway J. W., and Svejstrup J. Q. (2009) Distinct ubiquitin ligases act sequentially for RNA polymerase II poly-ubiquitylation. Proc. Natl. Acad. Sci. U.S.A. 106, 20705–20710 19920177 10.1073/pnas.0907052106 PMC2778569

[13] Kawauchi J., Inoue M., Fukuda M., Uchida Y., Yasukawa T., Conaway R. C., Conaway J. W., Aso T., and Kitajima S. (2013) Transcriptional properties of mammalian Elongin A and its role in stress response. J. Biol. Chem. 288, 24302–24315 23828199 10.1074/jbc.M113.496703 PMC3750133

[14] Petroski M. D., and Deshaies R. J. (2005) Function and regulation of cullin-RING ubiquitin ligases. Nat. Rev. Mol. Cell Biol. 6, 9–20 15688063 10.1038/nrm1547

[15] Mahrour N., Redwine W. B., Florens L., Swanson S. K., Martin-Brown S., Bradford W. D., Staehling-Hampton K., Washburn M. P., Conaway R. C., and Conaway J. W. (2008) Characterization of Cullin-box sequences that direct recruitment of Cul2-Rbx1 and Cul5-Rbx2 modules to Elongin BC-based ubiquitin ligases. J. Biol. Chem. 283, 8005–8013 18187417 10.1074/jbc.M706987200

[16] Weems J. C., Slaughter B. D., Unruh J. R., Hall S. M., McLaird M. B., Gilmore J. M., Washburn M. P., Florens L., Yasukawa T., Aso T., Conaway J. W., and Conaway R. C. (2015) Assembly of the Elongin A ubiquitin ligase is regulated by genotoxic and other stresses. J. Biol. Chem. 290, 15030–15041 25878247 10.1074/jbc.M114.632794 PMC4463447

[17] Boeing S., Williamson L., Encheva V., Gori I., Saunders R. E., Instrell R., Aygün O., Rodriguez-Martinez M., Weems J. C., Kelly G. P., Conaway J. W., Conaway R. C., Stewart A., Howell M., Snijders A. P., and Svejstrup J. Q. (2016) Multiomic analysis of the UV-induced DNA damage response. Cell Rep. 15, 1597–1610 10.1016/j.celrep.2016.04.047 PMC4893159 27184836

[18] Weidenheim K. M., Dickson D. W., and Rapin I. (2009) Neuropathology of Cockayne syndrome: evidence for impaired development, premature aging, and neurodegeneration. Mech. Ageing Dev. 130, 619–636 19647012 10.1016/j.mad.2009.07.006

[19] Newman J. C., Bailey A. D., and Weiner A. M. (2006) Cockayne syndrome group B protein (CSB) plays a general role in chromatin maintenance and remodeling. Proc. Natl. Acad. Sci. U.S.A. 103, 9613–9618 16772382 10.1073/pnas.0510909103 PMC1480455

[20] Wang Y., Chakravarty P., Ranes M., Kelly G., Brooks P. J., Neilan E., Stewart A., Schiavo G., and Svejstrup J. Q. (2014) Dysregulation of gene expression as a cause of Cockayne syndrome neurological disease. Proc. Natl. Acad. Sci. U.S.A. 111, 14454–14459 25249633 10.1073/pnas.1412569111 PMC4210037

[21] Vélez-Cruz R., and Egly J. M. (2013) Cockayne syndrome group B (CSB) protein: at the crossroads of transcriptional networks. Mech. Ageing Dev. 134, 234–242 23562425 10.1016/j.mad.2013.03.004

[22] Proietti-De-Santis L., Drané P., and Egly J. M. (2006) Cockayne syndrome B protein regulates the transcriptional program after UV irradiation. EMBO J. 25, 1915–1923 16601682 10.1038/sj.emboj.7601071 PMC1456931

[23] Troelstra C., van Gool A., de Wit J., Vermeulen W., Bootsma D., and Hoeijmakers J. H. J. (1992) *ERCC6*, a member of a subfamily of putative helicases, is involved in Cockaynes syndrome and preferential repair of active genes. Cell 71, 939–953 1339317 10.1016/0092-8674(92)90390-x

[24] van der Horst G. T. J., van Steeg H., Berg R. J. W., van Gool A. J., de Wit J., Weeda G., Morreau H., Beems R. B., van Kreijl C. F., de Gruijl F. R., Bootsma D., and Hoeijmakers J. H. J. (1997) Defective transcription-coupled repair in Cockayne syndrome B mice is associated with skin cancer predisposition. Cell 89, 425–435 9150142 10.1016/s0092-8674(00)80223-8

[25] Dianov G., Bischoff C., Sunesen M., and Bohr V. A. (1999) Repair of 8-oxoguanine in DNA is deficient in Cockayne syndrome group B cells. Nucleic Acids Res. 27, 1365–1368 9973627 10.1093/nar/27.5.1365 PMC148325

[26] Tuo J., Jaruga P., Rodriguez H., Bohr V. A., and Dizdaroglu M. (2003) Primary fibroblasts of Cockayne syndrome patients are defective in cellular repair of 8-hydroxyguanine and 8-hydroxyadenine resulting from oxidative stress. FASEB J. 17, 668–674 12665480 10.1096/fj.02-0851com

[27] Batenburg N. L., Thompson E. L., Hendrickson E. A., and Zhu X. D. (2015) Cockayne syndrome group B protein regulates DNA double-strand break repair and checkpoint activation. EMBO J. 34, 1399–1416 25820262 10.15252/embj.201490041 PMC4491999

[28] Tantin D., Kansal A., and Carey M. (1997) Recruitment of the putative transcription-repair coupling factor CSB/ERCC6 to RNA polymerase II elongation complexes. Mol. Cell. Biol. 17, 6803–6814 9372911 10.1128/mcb.17.12.6803 PMC232536

[29] van den Boom V., Citterio E., Hoogstraten D., Zotter A., Egly J. M., van Cappellen W. A., Hoeijmakers J. H., Houtsmuller A. B., and Vermeulen W. (2004) DNA damage stabilizes interaction of CSB with the transcription elongation machinery. J. Cell Biol. 166, 27–36 15226310 10.1083/jcb.200401056 PMC2172148

[30] Sarker A. H., Tsutakawa S. E., Kostek S., Ng C., Shin D. S., Peris M., Campeau E., Tainer J. A., Nogales E., and Cooper P. K. (2005) Recognition of RNA polymerase II and transcription bubbles by XPG, CSB, and TFIIH: insights for transcription-coupled repair and Cockayne Syndrome. Mol. Cell 20, 187–198 16246722 10.1016/j.molcel.2005.09.022

[31] Stevnsner T., Muftuoglu M., Aamann M. D., and Bohr V. A. (2008) The role of Cockayne Syndrome group B (CSB) protein in base excision repair and aging. Mech. Ageing Dev. 129, 441–448 18541289 10.1016/j.mad.2008.04.009 PMC2538557

[32] Sekar R. B., and Periasamy A. (2003) Fluorescence resonance energy transfer (FRET) microscopy imaging of live cell protein localizations. J. Cell Biol. 160, 629–633 12615908 10.1083/jcb.200210140 PMC2173363

[33] Van Munster E. B., Kremers G. J., Adjobo-Hermans M. J., and Gadella T. W. Jr. (2005) Fluorescence resonance energy transfer (FRET) measurement by gradual acceptor photobleaching. J. Microsc. 218, 253–262 15958019 10.1111/j.1365-2818.2005.01483.x

[34] Horibata K., Iwamoto Y., Kuraoka I., Jaspers N. G., Kurimasa A., Oshimura M., Ichihashi M., and Tanaka K. (2004) Complete absence of Cockayne syndrome group B gene product gives rise to UV-sensitive syndrome but not Cockayne syndrome. Proc. Natl. Acad. Sci. U.S.A. 101, 15410–15415 15486090 10.1073/pnas.0404587101 PMC524447

[35] Dinant C., de Jager M., Essers J., van Cappellen W. A., Kanaar R., Houtsmuller A. B., and Vermeulen W. (2007) Activation of multiple DNA repair pathways by sub-nuclear damage induction methods. J. Cell Sci. 120, 2731–2740 17646676 10.1242/jcs.004523

[36] Lukas C., Bartek J., and Lukas J. (2005) Imaging of protein movement induced by chromosomal breakage: tiny 'local' lesions pose great 'global' challenges. Chromosoma 114, 146–154 15988581 10.1007/s00412-005-0011-y

[37] Titov D. V., Gilman B., He Q. L., Bhat S., Low W. K., Dang Y., Smeaton M., Demain A. L., Miller P. S., Kugel J. F., Goodrich J. A., and Liu J. O. (2011) XPB, a subunit of TFIIH, is a target of the natural product triptolide. Nat. Chem. Biol. 7, 182–188 21278739 10.1038/nchembio.522 PMC3622543

[38] Jonkers I., Kwak H., and Lis J. T. (2014) Genome-wide dynamics of Pol II elongation and its interplay with promoter proximal pausing, chromatin, and exons. Elife 3, e02407 24843027 10.7554/eLife.02407 PMC4001325

[39] Menoni H., Hoeijmakers J. H., and Vermeulen W. (2012) Nucleotide excision repair-initiating proteins bind to oxidative DNA lesions *in vivo*. J. Cell Biol. 199, 1037–1046 23253478 10.1083/jcb.201205149 PMC3529521

[40] Iyama T., and Wilson D. M. 3rd (2016) Elements that regulate the DNA damage response of proteins defective in Cockayne syndrome. J. Mol. Biol. 428, 62–78 26616585 10.1016/j.jmb.2015.11.020 PMC4738086

[41] Gottschalk A. J., Timinszky G., Kong S. E., Jin J., Cai Y., Swanson S. K., Washburn M. P., Florens L., Ladurner A. G., Conaway J. W., and Conaway R. C. (2009) Poly(ADP-ribosyl)ation directs recruitment and activation of an ATP-dependent chromatin remodeler. Proc. Natl. Acad. Sci. U.S.A. 106, 13770–13774 19666485 10.1073/pnas.0906920106 PMC2722505

[42] Ahel D., Horejsí Z., Wiechens N., Polo S. E., Garcia-Wilson E., Ahel I., Flynn H., Skehel M., West S. C., Jackson S. P., Owen-Hughes T., and Boulton S. J. (2009) Poly(ADP-ribose)-dependent regulation of DNA repair by the chromatin remodeling enzyme ALC1. Science 325, 1240–1243 19661379 10.1126/science.1177321 PMC3443743

[43] Cleaver J. E., Thompson L. H., Richardson A. S., and States J. C. (1999) A summary of mutations in the UV-sensitive disorders: xeroderma pigmentosum, Cockayne syndrome, and trichothiodystrophy. Hum. Mutat. 14, 9–22 10447254 10.1002/(SICI)1098-1004(1999)14:1<9::AID-HUMU2>3.0.CO;2-6

[44] Anindya R., Mari P. O., Kristensen U., Kool H., Giglia-Mari G., Mullenders L. H., Fousteri M., Vermeulen W., Egly J. M., and Svejstrup J. Q. (2010) A ubiquitin-binding domain in Cockayne syndrome B required for transcription-coupled nucleotide excision repair. Mol. Cell 38, 637–648 20541997 10.1016/j.molcel.2010.04.017 PMC2885502

