# Supplementary material for: Cockayne syndrome B protein regulates recruitment of the Elongin A ubiquitin ligase to sites of DNA damage
Source: J Biol Chem. 2017 Mar 14;292(16):6431–7. doi: 10.1074/jbc.C117.777946 (PMC5399097; doi:10.1074/jbc.C117.777946)
Supplement: Supplemental Data [file supp_292_16_6431__index.html]

Cockayne Syndrome B Protein Regulates Recruitment of the Elongin A Ubiquitin Ligase to Sites of DNA Damage — Cockayne syndrome B protein regulates recruitment of the Elongin A ubiquitin ligase to sites of DNA damage — ACCELERATED COMMUNICATION: CSB-dependent recruitment of Elongin A — Supplemental Data 

# Cockayne syndrome B protein regulates recruitment of the Elongin A ubiquitin ligase to sites of DNA damage

## Supplemental Data

- Supplemental Figures (.pdf, 858 KB) - Supplemental Figure 1. CSB-dependent recruitment of Elongin A (A) and CUL5 (B) to localized DNA damage in CS1ANsv cells. Supplemental Figure 2. AP-FRET between wild type or mutant Halo-Elongin A labeled with TMRDirect and GFP-CSB (A) or Halo-Elongin A labeled with rhodamine 110 and mCherry-CUL5 (C). B. Kinetics of recruitment of wild type and mutant Halo-Elongin A.
