# Supplementary material for: Cockayne syndrome B protein regulates recruitment of the Elongin A ubiquitin ligase to sites of DNA damage
Source: J Biol Chem. 2017 Mar 14;292(16):6431–7. doi: 10.1074/jbc.C117.777946 (PMC5399097; doi:10.1074/jbc.C117.777946)

Supplemental Material

Cockayne Syndrome B Protein Regulates Recruitment of the Elongin A Ubiquitin Ligase to Sites of DNA Damage

Juston C. Weems[1], Brian D. Slaughter[1], Jay R. Unruh[1], Stefan Boeing[2], Shawn M. Hall[1], Merry B. McLaird[1], Takashi Yasukawa[4], Teijiro Aso[4], Jesper Q. Svejstrup[2], Joan W. Conaway[1, 3] and Ronald C. Conaway[1, 3]

Supplemental Figure Legends

Supplemental Figure 1. CSB-dependent recruitment of Elongin A (*A*) and CUL5 (*B*) to localized DNA damage in CS1ANsv cells, transiently transfected (red) or not (black) with GFP-CSB. Recruitment of GFP-CSB is shown in blue. Graphs show mean ± SEM, n=18 cells (6 cells from each of 3 independent experiments). Arrows in graphs indicate time of microirradiation; white triangles in images indicate microirradiated regions. Scale bars, 8 µm.

Supplemental Figure 2. AP-FRET between wild type or mutant Halo-Elongin A labeled with TMRDirect and GFP-CSB (*A*) or Halo-Elongin A labeled with rhodamine 110 and mCherry-CUL5 (*C*). The graphs show individual data points, median, and interquartile ranges obtained from AP-FRET measurements made in a total of 24 cells for each FRET pair (6 cells from each of 4 independent experiments). *B*. Kinetics of recruitment of wild type and mutant Halo-Elongin A (6 cells each from 4 independent experiments). Cells were imaged every second, and intensity values were binned over 5-s intervals. Microirradiation was initiated at time t=0 s. Values represent mean ± SEM.

Supplemental Figure 1

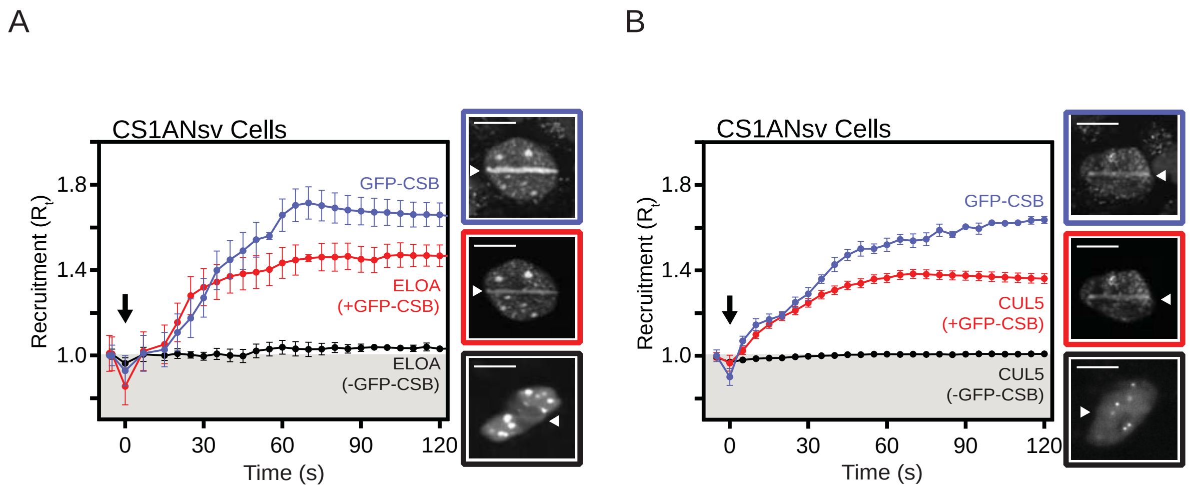

Supplemental Figure 2

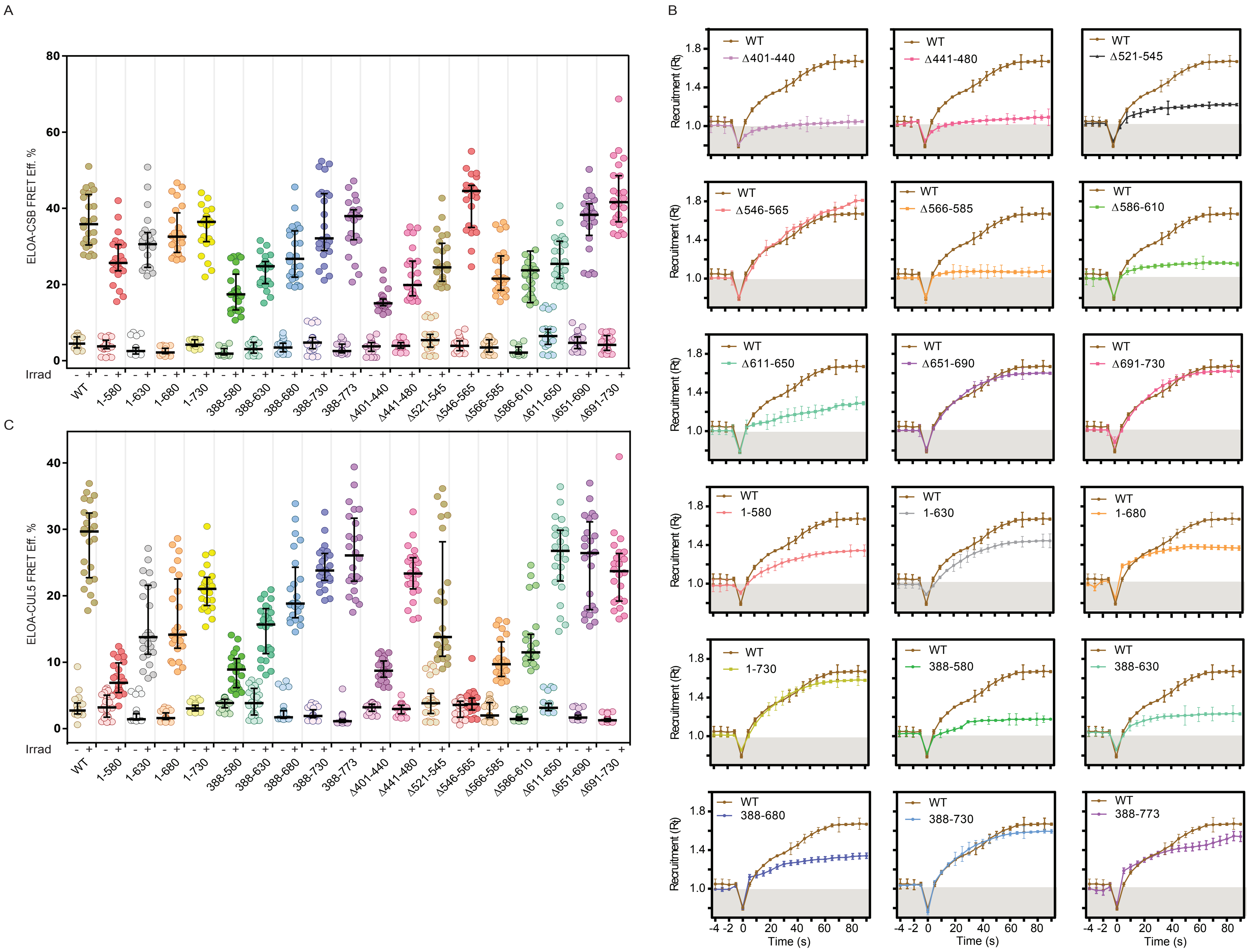

Supplement: Supplemental Data [file 10.1074_C117.777946_jbc.C117.777946-1.pdf]
